# Influenced but not determined by historical events: genetic, demographic and morphological differentiation in *Heleobia ascotanensis* from the Chilean Altiplano

**DOI:** 10.7717/peerj.5802

**Published:** 2018-12-17

**Authors:** Moisés A. Valladares, Marco A. Méndez, Gonzalo A. Collado

**Affiliations:** 1 Laboratorio de Genética y Evolución, Departamento de Ciencias Ecológicas, Facultad de Ciencias, Universidad de Chile, Santiago, Región Metropolitana, Chile; 2 Departamento de Ciencias Básicas, Facultad de Ciencias, Universidad del Bío-Bío, Chillán, Región del Bío-Bío, Chile

**Keywords:** Microgeographic differentiation, Morphometric analysis, Phylogeography, Freshwater snails, Humid-arid transitions

## Abstract

In the present study, we focus on the phylogeographic pattern, demographic history and morphological differentiation of *Heleobia ascotanensis*, a freshwater gastropod restricted to the Ascotán saltpan in the Chilean Altiplano. The current distribution of the species is limited to twelve isolated or partially isolated springs that were affected by transitions between humid and arid periods during last glaciations. The genetic analysis of 322 specimens showed that *H. ascotanensis* is subdivided into three genetically divergent populations, with low and moderate degrees of historical gene flow among them and incipient morphological differentiation as a consequence of genetic and geographical isolation. Molecular analyses revealed different demographic histories among populations which seem to respond independently to climatic events, probably due to an environmental imposition and idiosyncratic strategies developed to cope with water availability. The results of this study and co-distributed taxa support the hypothesis that contemporary and historical events have influenced microevolutionary differentiation of these snails, although there is a need to complement further information to predict genetic or morphological divergence at microgeographic scale.

## Introduction

The physiography of the South American Altiplano landscape is characterized by several closed basins mainly originated by a high volcanic activity that took place since the Miocene, accompanied by complex climatic events (Fornari, Risacher & Féraud, 2001; Risacher, Alonso & Salazar, 2003; Strecker et al., 2007; Placzek, Quade & Patchett, 2011; Placzek, Quade & Patchett, 2013). At present, the internal hydrological systems constitute closed evaporitic ecosystems with unique properties transforming the region in a natural scenery for the study of the evolutionary diversification of aquatic biota (Collado, Valladares & Méndez, 2013; Vila et al., 2013; Victoriano et al., 2015). Nowadays the Altiplano is characterized by having conditions of aridity during most of the year, except for humid period known as South American Summer Monsoon (SASM) from December to March (Zhou & Lau, 1998; Garreaud et al., 2009; Marengo et al., 2012). The SASM is characterized by intense convective activity and precipitation that reach its maximum during the austral summer (Carvalho et al., 2011; Carvalho & Cavalcanti, 2016), and its year-to-year variability in precipitation is mainly associated to changes in the mean zonal wind over the Altiplano, largely modulated by sea surface temperature (SST) across the tropical Pacific Ocean (Vuille, Bradley & Keimig, 2000; Garreaud & Aceituno, 2001). Regarding variability in a temporal climatic context, the SASM showed a coherent regional signal of a weak monsoon during the Medieval Climate Anomaly period (*c.* 950–1250) and a stronger event during the Little Ice Age (*c.* 1450–1850) (Carvalho et al., 2011). Contrasting the actual hydrographic conditions on the Altiplano mainly modulated by the SASM, during the Pleistocene most of the region was covered by great paleolakes over 3.650 m altitude (influenced by global glaciations), which connected numerous hydrographic basins currently disconnected (Lavenu, Fornari & Sebrier, 1984; Fornari, Risacher & Féraud, 2001; Blard et al., 2011; Placzek, Quade & Patchett, 2013). With the transition of humid periods that characterized the Plio-Pleistocene to dry seasons prevailing during the Holocene, the aquatic environments were fragmented causing the isolation of populations confined to these systems (e.g.,  Keller & Soto, 1998).

Taxa distributed in patchy aquatic systems show highly geographically structured populations, probably because appropriate habitats are often surrounded by inappropriate environments, therefore gene flow usually depends on the dispersal capabilities of the species (Johnson, 2005). The genetic structure of freshwater fauna confined to the Altiplano usually reflects environmental changes that fragmented previously continuous hydrological systems, changes particularly important for poorly dispersing species such as snails and amphipods (Seidel, Lang & Berg, 2009; Murphy et al., 2012). This pattern has been demonstrated at species and population level in species of snails (Collado, Vila & Méndez, 2011; Collado & Méndez, 2012a; Collado & Méndez, 2013), fishes (Keller & Soto, 1998; Morales, Vila & Poulin, 2011; Cruz-Jofré et al., 2016; Guerrero-Jiménez et al., 2017) and amphibians (Sáez et al., 2014; Victoriano et al., 2015).

A large number of species of small caenogastropods of the genus *Heleobia* Stimpson, 1865 inhabit a variety of brackish and freshwater ecosystems in the Andean Altiplano (Courty, 1907; Pilsbry, 1924; Biese, 1944; Biese, 1947; Haas, 1955; Hubendick, 1955; Dejoux & Iltis, 1992; Collado, 2015; Collado, Valladares & Méndez, 2013; Collado, Valladares & Méndez, 2016). Given the similarity in external shell morphology, shell traits (the only ones available in some of them) become inconsistent at the time of identifying and delimiting species in the group (Cazzaniga, 2011; Collado, Valladares & Méndez, 2013), although morphometric analyses have detected some morphological differentiation among different lineages (Collado, Valladares & Méndez, 2016). The Ascotán saltpan is located in the southern Altiplano in Chile and is the type locality of *Heleobia ascotanensis* (Courty, 1907), a species comprising five subspecies that were described based uniquely on external shell morphology (Courty, 1907); the original description by Courty indicates that in the saltpan, besides *Heleobia*, originally described under the genus *Paludestrina* d’Orbigny, 1840, dwell two species of the genus *Bithinella* Fischer, 1885 (see Collado & Méndez, 2012b). Subsequent studies have shown that only *Heleobia* specimens are found in the saltpan, therefore Courty (1907) probably overestimated the number of species in the system due to subtle morphological differences among the individuals analysed (Collado, Valladares & Méndez, 2013; Collado, Valladares & Méndez, 2016). Previous molecular phylogenetic studies have showed that *Heleobia* populations from this saltpan are genetically differentiated from the rest of populations inhabiting the Altiplano (Collado, Valladares & Méndez, 2013), as well as suggested the existence of some genetic structure within the system. Moreover, the monophyly and support values suggest that two distinct lineages co-occur in the Ascotán saltpan (Collado, Valladares & Méndez, 2013; Collado, Valladares & Méndez, 2016). It is also important to note that the saltpan comprises twelve isolated or partially isolated springs and both existing molecular studies that analysed *Heleobia* populations from the Ascotán saltpan, only included two springs so the genetic diversity could be subestimated.

The pulmonate freshwater snail *Biomphalaria crequii* (Courty, 1907) is co-distributed with *Heleobia* in a large part of the Ascotán saltpan*.* A genetic study carried out in this system showed that three genetic groups were present within *B. crequii* populations, one of them restricted to the north-central area of the saltpan, a second including snails from the south-central section, and a third from the southernmost spring (Collado & Méndez, 2013). Similarly, another molecular study revealed that the endemic killifish *Orestias ascotanensis* Parenti, 1984 presents high levels of genetic diversity that accounts for four differentiated groups, two located at the north and south ends and other two including several springs confined to the central area of the system being indicative of some gene flow among populations (Morales, Vila & Poulin, 2011; Cruz-Jofré et al., 2016). It has been suggested that this pattern of genetic structure in these taxa was mainly stimulated by a combination of factors such as fluctuation of water levels (historical and contemporary), different altitude ranges and geographic isolation of populations (see Morales, Vila & Poulin, 2011; Collado & Méndez, 2013; Cruz-Jofré et al., 2016).

In the present study, we examined the phylogeographic and demographic history of *H. ascotanensis* from the Ascotán saltpan to test whether this species shows population structure and whether this structure is associated with the physiography of the saltpan or it responds to past climatic events. Also, we estimated the historical gene flow among populations inhabiting the different springs of the system. Finally, we analysed morphological data to test for congruent variation between genetic groups. We hypothesized that *H. ascotanensis* would show high genetic structure due to geographical and environmental influences, particularly associated to the last three major humid periods in the region (Sajsi [*c.* 21–25 kya], Tauca [*c.* 14–18 kya] and Coipasa [*c.* 11–13 kya]). Ultimately, we proposed that the microevolutionary differentiation would be similar to the patterns described in co-distributed taxa, but not identical, reflecting an idiosyncratic response to the same historical events.

## Study Area

**Figure 1:**
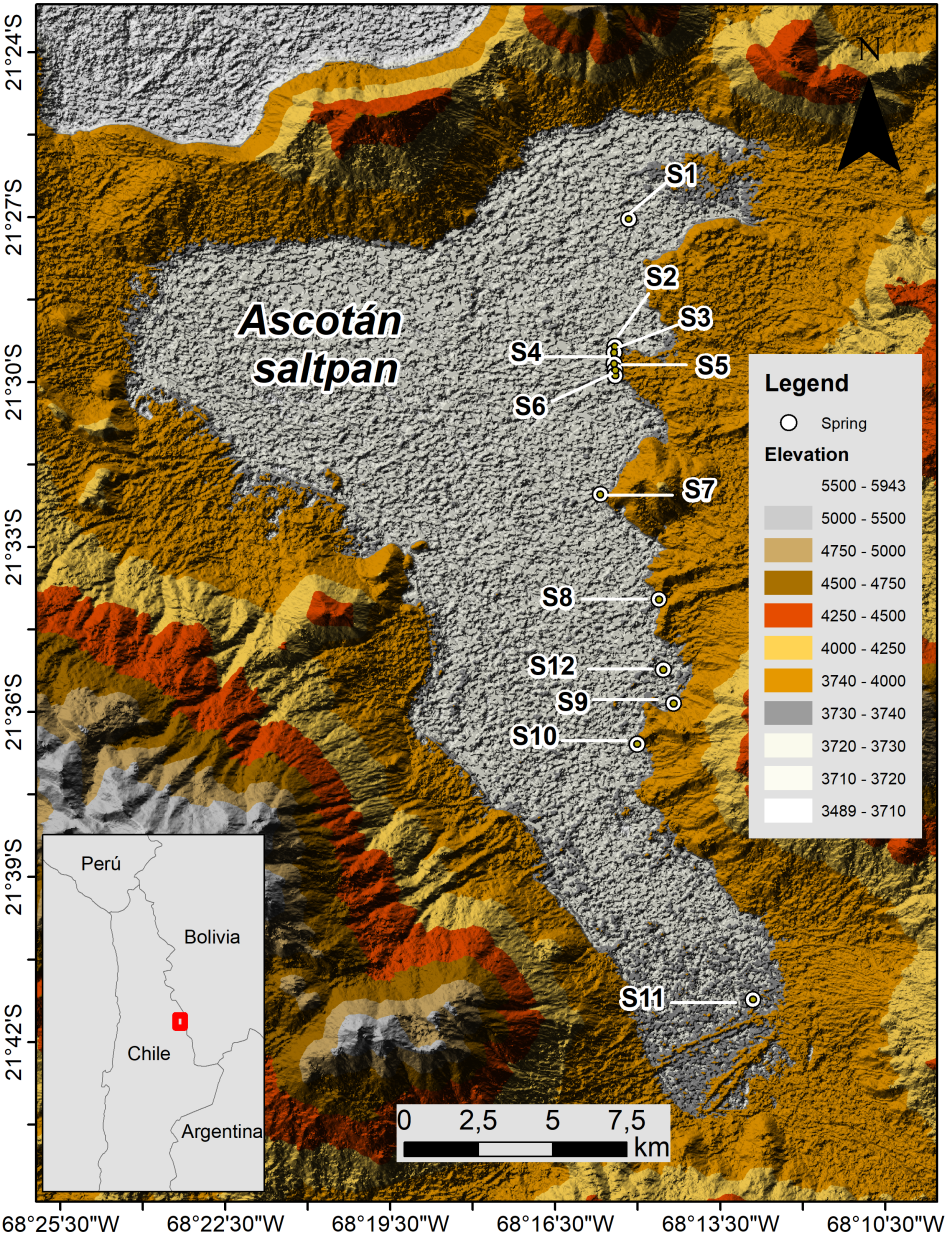
Map of Ascotán saltpan. The twelve sampled localities (springs, denoted by S) are showed. Lighter colors indicate lower altitude ranges.

The Ascotán saltpan is one of the closed basins located in the Chilean Altiplano (Región de Antofagasta) between 21.2–21.4°S and 68.1–68.2°W at 3,760 m a.s.l. The hydrological system extends around 33 km long by 16 km wide at its maximum range, representing a surface which is mainly covered by evaporitic deposits (243 km^2^), whose main water body (18 km^2^) is generated by a series of springs located mainly in the eastern area of the system (Fig. 1). These springs are fed by underground water coming from the contiguous basin Pastos Grandes Caldera located at 4,600 m altitude (Keller & Soto, 1998; Risacher, Alonso & Salazar, 1999; Risacher, Alonso & Salazar, 2003). On the eastern margin, twelve springs are isolated or partially isolated by vast areas of evaporitic lands that constitute topographic barriers difficult to cross for the aquatic organisms, so connectivity among springs and populations that inhabiting them is reduced. These springs are considered thermal since the water temperature varies among 16–22 ° C, and the high conductivity and salinity indicates the ionic nature of the saltpan (Keller & Soto, 1998; Márquez-García et al., 2009; Risacher, Fritz & Hauser, 2011).

## Material & Methods

### Sample collection

The specimens of *H. ascotanensis* were collected in the water body in two periods due to climatic conditions that did not allow us to sample all springs at once. In April 2012 all springs (except Spring 1) were sampled, and in April 2013 Spring 1 was visited. Also, in April 2013 we collected additional specimens from Spring 11 and Spring 12, these individuals share the same genetic pool (haplotypes) and morphometric features as the specimens collected from the corresponding springs the year before, therefore they were considered as the same population. A minimum of 60 snails were collected by hand or using a small sieve (1 mm mesh) from sediment or macrophytes (usually *Lilaeopsis macloviana* (Gand.) A.W. Hill and *Ruppia filifolia* (Phil.)) in each spring. Sampled specimens were preserved in absolute ethanol for posterior morphological and genetic analyses. Gonzalo A. Collado was authorized to collect *Heleobia* snails in Chile (Resolution No. 3285, Subsecretaría de Pesca y Acuicultura, Ministerio de Economía, Fomento y Turismo, República de Chile).

### Molecular methods

Genomic DNA was extracted from specimens using the cetyl trimethyl ammonium bromide (CTAB) method (Winnepenninckx, Backeljau & De Wachter, 1993). Partial sequences of the cytochrome oxidase subunit I (COI) gene were amplified by the polymerase chain reaction using the primers HCO2198 and LCO1490 (Folmer et al., 1994). We used this mitochondrial gene for its usefulness in discriminating species through genetic barcode (Hebert et al., 2003), wide application in phylogeographic (and even phylogenetic) studies (Wilke, Schultheiß& Albrecht, 2009; Kroll et al., 2012; Wilke et al., 2013), and easy amplification, sequencing and analysis. Both strands of the amplified products were Sanger-sequenced at Macrogen Inc., Seoul, Korea. Forward and reverse strands were corrected for misreads and merged into one sequence file using CODONCODE ALIGNER v3.6.1 (CodonCode Corporation, Dedham, MA, USA).

### Phylogeographic and genetic analyses

We calculated the number of polymorphic sites (S), number of haplotypes (K), haplotypic diversity (H) and nucleotide diversity (*π*) in DnaSP (Librado & Rozas, 2009). To visualise the relationships between haplotypes, we constructed a median-joining network (Bandelt, Forster & Röhl, 1999) using the program PopART (Leigh & Bryant, 2014) with ε = 0. Pairwise *F*_ST_ values were calculated in ARLEQUIN v3.5 (Excoffier, Laval & Schneider, 2005) to investigate the degree of genetic differentiation among individuals sorted by springs (significance determined by 10,000 permutations). A Bayesian analysis of population structure accounting for the geographical distribution of *H. ascotanensis* was performed with the GENELAND v4.0.6 package (Guillot, Mortier & Estoup, 2005) in R v3.4.1 (R Core Team, 2017). GENELAND incorporates geographical coordinate information for genotyped individuals to estimate the most likely number of genetic groups or clusters (CT) and their spatial boundaries. For all GENELAND analyses we used an uncorrelated allele frequencies model, considering that an isolation-by-distance (IBD) pattern can cause algorithm instabilities in the correlated model. We also set the coordinate uncertainty to 0.0001 to allow for the possibility of different individuals captured at the same point to be assigned to different populations (Guillot, 2008). Our data set was analysed over 15 independent runs, assuming a CT_min_ = 1 and a CT_max_ = 12 (total number of springs) and using the Dirichlet model. Analyses were run using 10^8^ MCMC iterations and collecting data at every 100th iteration (see Guillot, 2008). We tested for hierarchical population structure with an analysis of molecular variance (AMOVA) using ARLEQUIN. To test for local structure at the population level, we placed all specimens from the twelve springs analysed into two different hypothesized population groupings according to the genetic clusters determined by the GENELAND package and the haplogroups defined by the median-joining network. Historical demography was studied by comparing the observed mismatch distributions with those expected under a sudden expansion model (Rogers & Harpending, 1992) using the sum of the squared deviations (SSD) implemented in ARLEQUIN and 10,000 bootstrap replicates to assess significance. Harpending’s Raggedness index (rH) was also calculated to test the unimodality of observed data. ARLEQUIN was also used to calculate the Tajima’s D (Tajima, 1989) and Fu’s FS indices (Fu, 1997), which are sensitive to demographic changes.

To estimate the tendency of population growth through time we constructed a Bayesian skyline plot (BSP) as implemented in BEAST2 (Bouckaert et al., 2014) on XSEDE via the Cyberinfrastructure for Phylogenetic Research Science (CIPRES) gateway portal (Miller, Pfeiffer & Schwartz, 2010). This Bayesian approach incorporates uncertainty in genealogy by using MCMC integration under a coalescent model, providing information about effective population sizes (Ne) through time (Drummond et al., 2005). The best-fitting substitution model was estimated in bModelTest (Bouckaert & Drummond, 2015). The running conditions included 10^7^ iterations sampling model parameters every 1,000 steps using a relaxed exponential clock model and assuming a substitution rate of 1.7% substitutions per million years for invertebrates (Wilke, Schultheiß& Albrecht, 2009). The first 25% of steps were discarded to allow for burn-in. To assess the robustness of parameter estimates, two independent chains were run with identical settings. Log files were visualized using TRACER 1.6 (Rambaut, Suchard & Drummond, 2014).

The PAST3 software (Hammer, Harper & Ryan, 2001) was used to perform Mantel tests to evaluate the existence of isolation-by-distance (IBD). For this analysis, we determined the significance of correlations between matrices of pairwise distances between populations setting 10,000 randomizations, using the genetic distance matrix (*F*_ST_) and geographical distances matrix between springs. We estimated the magnitude and direction of historic gene flow among spring populations grouped according to the results of the genetic structure analysis (haplogroups and genetic clusters), using MIGRATE-n v3.2.7 (Beerli, 2006) on CIPRES. Estimations of past mutation-scaled migration rates (*M*) among the studied populations were performed by grouping the individuals according to haplogroups and genetic clusters (defined by the GENELAND analysis). For the analysis, a Brownian motion model was run to estimate *M*, where *M* = *m*∕*μ* (being *m* the historical migration and *μ* the mutation rate). The parameters (Φ) (mutation-scaled population size) and *M* followed an exponential distribution (0–100, mean = 50; 0–1,000, mean = 100, respectively), heating was set with four temperatures (1.0, 1.5, 3.0 and 10,000) with a static scheme. We ran 10 short chains with a total of 10,000 genealogy samples and three long chains with 1,000,000 samples, following a burn-in of 1,000 samples; three independent runs were performed.

### Morphometric analyses

We studied shell shape variation of *Heleobia* populations from the Ascotán saltpan using outline analyses based on the elliptic Fourier analysis (EFA). To avoid issues associated with developmental size changes, we exclude juveniles from the analysis. Since we were unable to determine which specimens had reached maturity from shell characters by themselves, we used size as a proxy, analysing specimens in the upper third of the size distribution. Samples used in morphometric analyses (*N* = 322) corresponded to the same samples used in the molecular analysis. Since genetic analyses results were partially discordant, three different datasets were analysed: (i) the first dataset contained individuals organized by its spring of origin; (ii) the second dataset contained individuals sorted by haplogroups and; (iii) the third dataset contained individuals pooled by genetic clusters defined by GENELAND analysis.

Regarding the morphological study, the image of the shell of at least 23 snails per spring (Table 1) was taken from a ventral view using a camera (Moticam 5) coupled to a stereomicroscope (Motic SMZ-168). The images were subsequently processed in Adobe Photoshop CS6 (Adobe Systems Inc, 2012) to create a black-and-white bitmap image. Elliptic Fourier transformations were made using SHAPE software (Iwata & Ukai, 2002). With the software ChainCoder, we extracted the contours of the objects and the relevant information was stored as chain codes. We obtained the normalized EFDs from the chain-coded contour with the software Chc2Nef, and the coefficients of the EFDs were calculated by discrete Fourier transformation following Kuhl & Giardina (1982). We estimated that 30 harmonics were sufficient to reconstruct the outlines with high exactitude and resolution these 30 harmonics represent 99.99% of the total Fourier power spectrum (Crampton, 1995). We performed a principal components analyses on the variance–covariance matrix of the EFD coefficients in the software PrinComp. The seven effective principal components (number of PC whose proportion is larger than 1/Number of analysed coefficients) that represented 94.54% of the total variance were used for the subsequent analyses. Shape differences between individuals from different springs and haplogroups were tested using a non-parametric multivariate analysis of variance (PERMANOVA) using 10,000 permutation in PAST3. Multivariate normality was checked with a multivariate normality test implemented in PAST3 that computes Mardia’s multivariate skewness and kurtosis, with tests based on chi-squared (skewness) and normal (kurtosis) distributions. To test if the groups are morphologically distinct and to observe the percentage of correctly classified individuals in each group, linear discriminant analysis (LDA) was performed in PAST3. In addition, group assignment was cross-validated by leave-one-out cross-validation (jackknifing) procedure.

**Table 1:** Description of sampling springs of *Heleobia ascotanensis* specimens. Number of individuals per spring and their respective diversity indices based on mtDNA (COI) sequences.

Spring	Geographical coordinates	Altitude (m)	*N*	Number of polymorphic sites	Number of haplotypes	Haplotyope diversity	Nucleotide diversity	Mean number of pairwise differences
1	21°27′02.0″S 68°15′09.8″W	3,733	29	15	8	0.527	0.00362	2.138
2	21°29′21.2″S 68°15′24.8″W	3,732	41	11	5	0.659	0.00837	4.949
3	21°29′27.8″S 68°15′25.6″W	3,734	23	12	5	0.723	0.00725	4.285
4	21°29′39.8″S 68°15′25.6″W	3,730	27	14	6	0.692	0.00901	5.328
5	21°29′47.2″S 68°15′24.5″W	3,729	24	10	4	0.634	0.00880	5.199
6	21°29′52.8″S 68°15′24.4″W	3,732	25	10	4	0.657	0.00866	5.120
7	21°32′02.7″S 68°15′40.7″W	3,731	24	5	3	0.562	0.00145	0.855
8	21°33′57.2″S 68°14′36.8″W	3,738	24	6	4	0.511	0.00143	0.848
9	21°36′11.7″S 68°15′01.4″W	3,735	23	6	3	0.561	0.00161	0.949
10	21°36′35.0″S 68°15′00.7″W	3,738	24	4	5	0.634	0.00138	0.815
11	21°41′13.9″S 68°12′54.0″W	3,740	34	11	13	0.822	0.00284	1.676
12	21°35′13.9″S 68°14′32.3″W	3,735	24	3	4	0.562	0.00107	0.634
Total			322	32	36	0.810	0.00740	4.376

## Results

### Population genetic and demographic analysis

The amplification of the COI gene produced a fragment of 591 nucleotides. The nucleotide composition in the complete dataset was 23.7% A, 17.2% C, 19.3% G and 39.7% T. Of all specimens examined, the sequence alignment showed a total of 32 polymorphic and 26 parsimony informative sites, defining 36 haplotypes (GenBank accession numbers: MF447901–MF448222). The best-fitting model of nucleotide substitution, as determined bModelTest, was the General Time Reversible model plus invariable sites (GTR+I), with *I* = 0.81. The mtDNA COI sequences of *Heleobia* showed a pattern of moderate haplotype diversity (*H* = 0.810), but high nucleotide diversity (*π* = 0.0074). At the spring level, Spring 11 presented the highest value of haplotype diversity and the highest value of nucleotide diversity was found in Spring 4. Of the remaining springs, the lowest value of haplotype diversity was detected in Spring 8 and the lowest value for nucleotide diversity in Spring 12 (Table 1).

**Figure 2:**
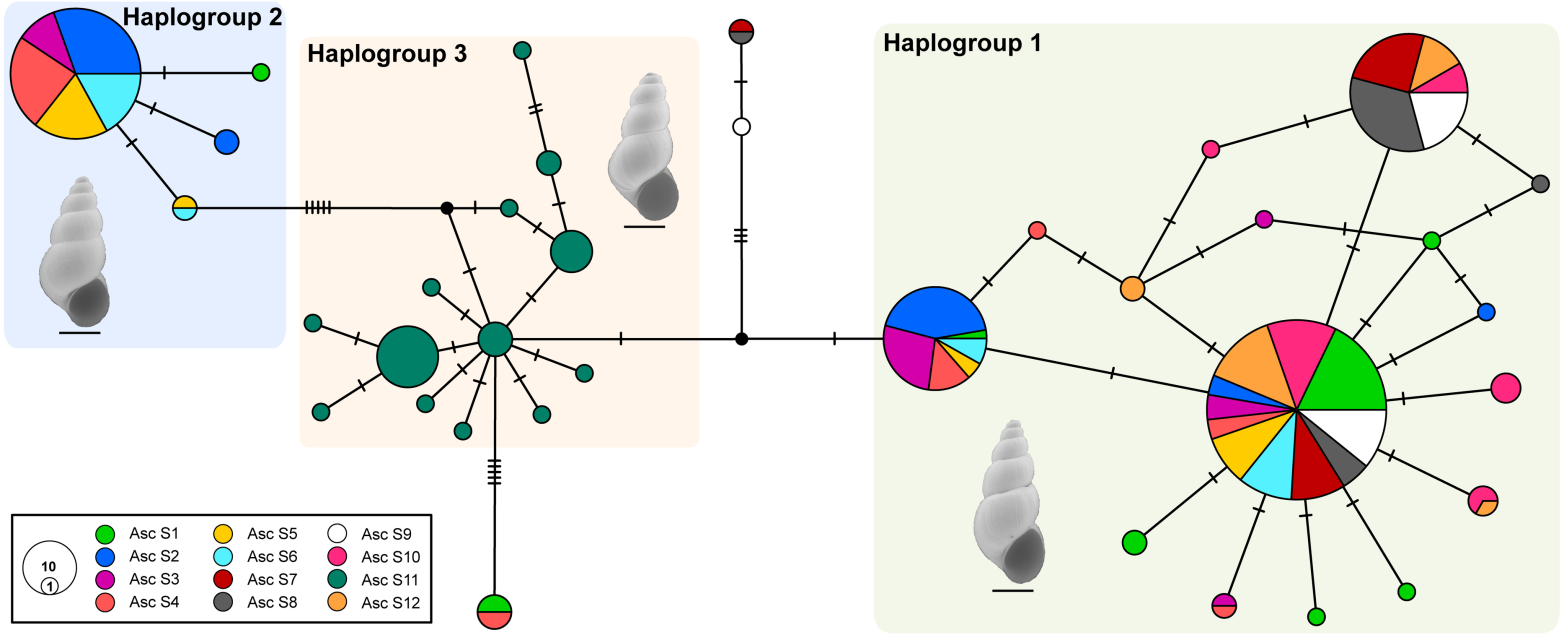
Haplotype network. Genealogical relationships of the mtDNA haplotypes found in *H. ascotanensis*. Circle sizes are proportional to haplotype frequencies and colors represent the respective sample localities. Representative snails are depicted along each haplogroup (scale bar: 1 mm).

**Figure 3:**
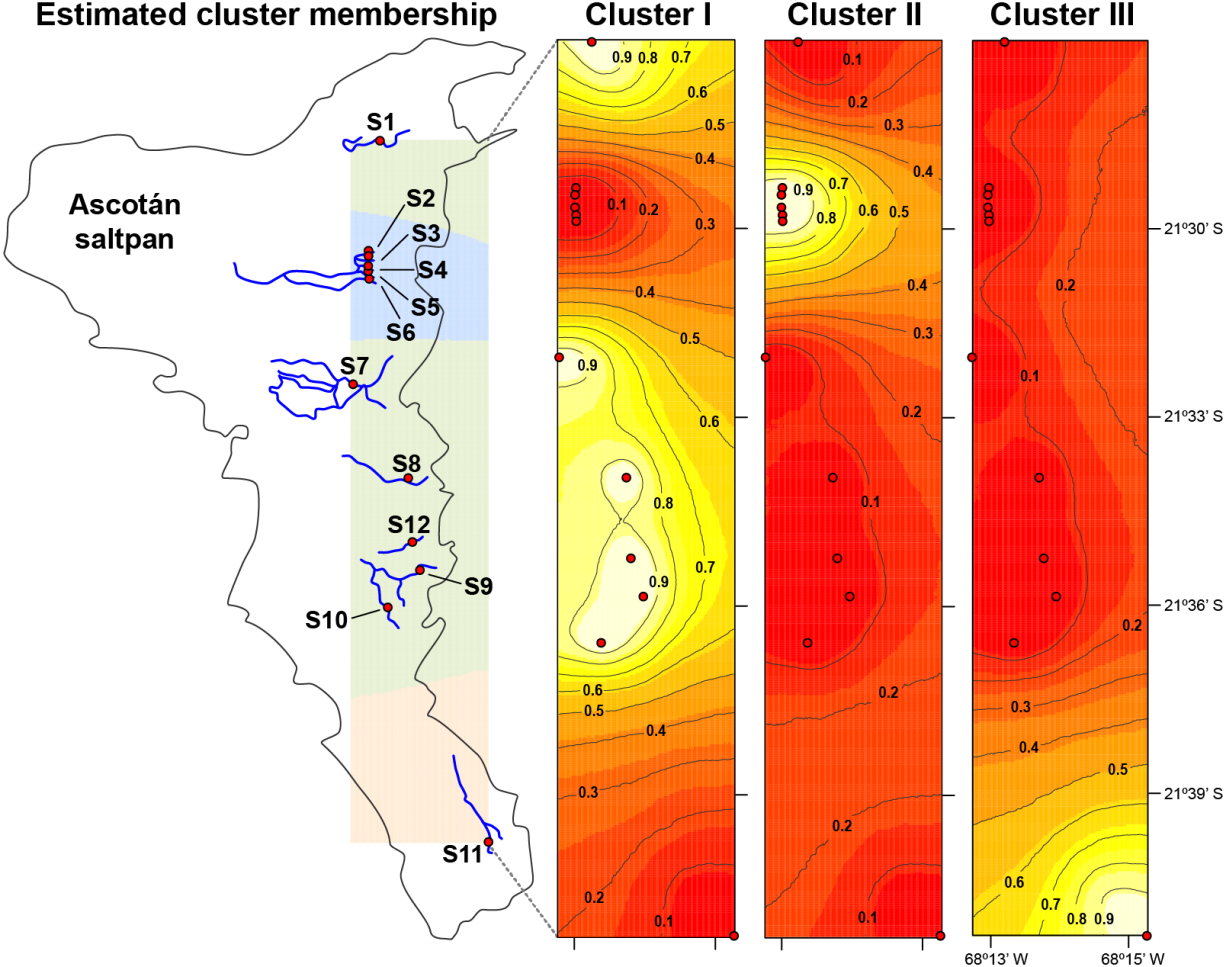
Population structure in *H. ascotanensis.* GENELAND analysis with estimated cluster membership and posterior probability. Lighter shading indicates higher probabilities of population membership.

The median-joining network recovered three well-differentiated haplogroups (Fig. 2). One group (Haplogroup 1), comprising haplotypes found in almost all the springs (except Spring 11), showed a star-like structure centred on the haplotype with the highest frequency (112 samples) spread over eleven springs. The second haplogroup (Haplogroup 2), which recovered sequences from Spring 2 to Spring 6 (and one sequence from Spring 1), showed a star-like structure integrated by four haplotypes. The third group (Haplogroup 3) included all specimens from Spring 11, the southernmost spring of the system. In this haplogroup, a star-like shape of the network was less apparent. The overall differentiation between populations was very high (*F*_ST_ = 0.535), and the closest springs showed lower values of differentiation when compared to remote springs (Table  S1), indicating that almost all genetic variation lies among populations. The population structure estimated through GENELAND analysis generated three genetic clusters (CT = 3), called Clusters I, II, and III (Fig. 3). Each population inferred had a posterior probability of 0.90 belonging to one of the assumed clusters, providing strong support to the GENELAND analysis results. Cluster I comprised all samples from Spring 1, Spring 7 to Spring 10, and Spring 12, while Cluster II was represented by populations of Spring 2 to Spring 6. Cluster III comprised only the population from Spring 11. The AMOVA identified significant values for the genetic variance at the two levels tested (among haplogroups defined by median-joining network and among the genetic clusters determined by GENELAND analysis). Grouping populations by haplogroups explained more of the genetic variation (86.67%, *P* <  0.005; Table 2) than grouping populations by GENELAND analysis (45.13%, *P*  <  0.005; Table 2), probably indicating that GENELAND analysis grouped genetically distant individuals into the same genetic cluster due to geographical proximity.

**Table 2:** Analysis of molecular variance (AMOVA). Results evaluating two classifications (haplogroups and genetic clusters). Results are expressed as the percentage of total variation.

Group level	Source of variation	*d*.*f*.	Sum of squares	Variance components	Percentage of variation (%)	Fixation index (*P* < 0.005)
Haplogroups	Among Haplogroup 1, 2 and 3	2	536.376	3.537	86.67	ΦCT = 0.867
	Among populations within haplogroups	12	35.164	0.114	2.79	ΦSC = 0.209
	Within populations	300	130.739	0.430	10.54	ΦST = 0.895
GENELAND clusters	Among Cluster I, II and III	2	236.130	1.214	45.13	ΦCT = 0.451
	Among populations within clusters	7	25.289	0.053	1.99	ΦSC = 0.036
	Within populations	305	440.860	1.422	52.88	ΦST = 0.471

**Figure 4:**
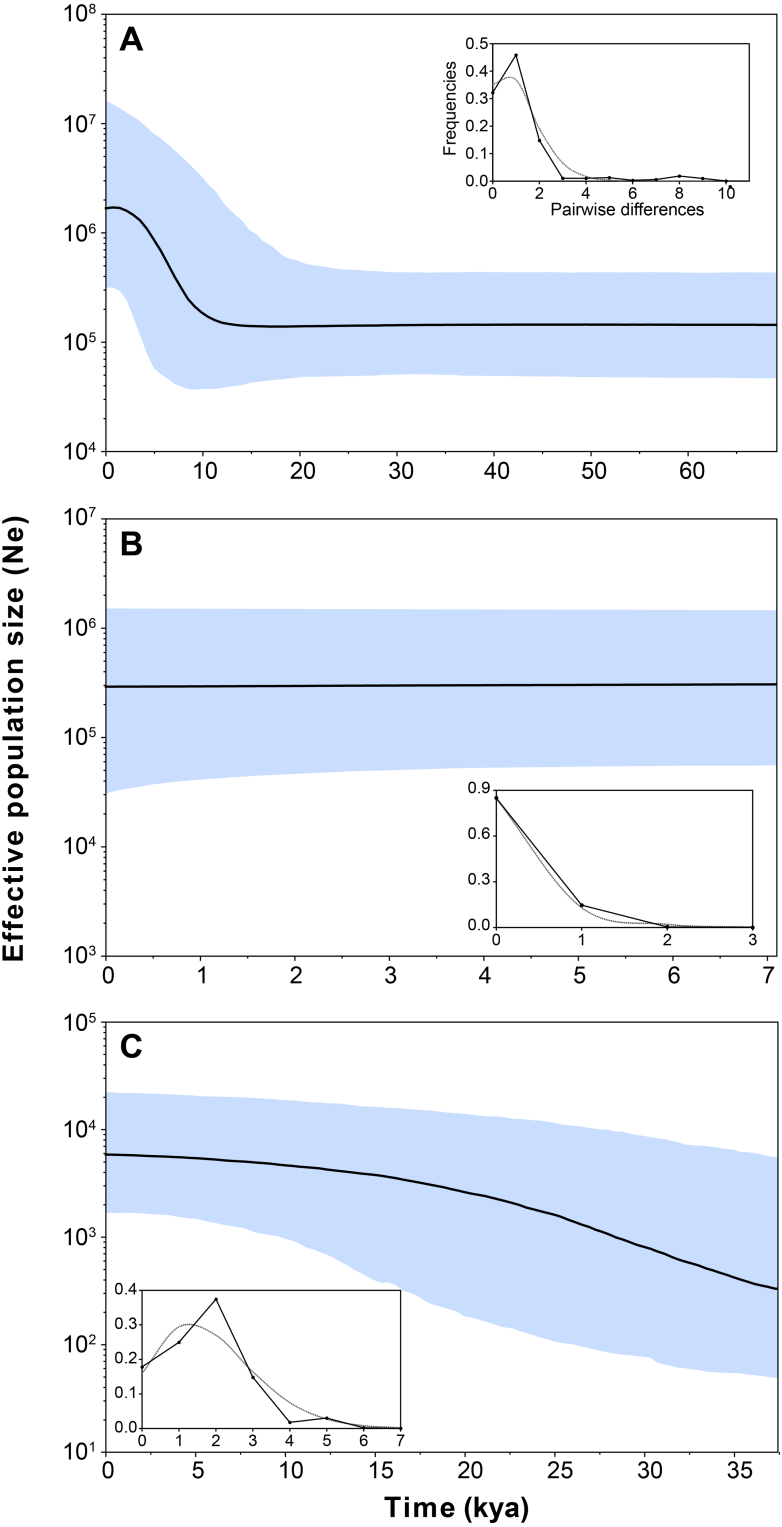
Bayesian skyline plot and mismatch distributions (inset graphs) depicting the demographic history for *H. ascotanensis* retrieved haplogroups. (A) Haplogroup 1. (B) Haplogroup 2. (C) Haplogroup 3. For the skyline plot, black lines represent median estimates, whereas the shaded area represent the 95% credible interval. For mismatch distributions, black dots represent the observed distribution of pairwise differences and gray line represent the theoretical expected distribution under a population expansion model.

Demographic analyses found a consistent signature of recent population expansion in Haplogroup 1, and an old expansion in Haplogroup 3 (Spring 11). Probably due to the extremely low genetic diversity in Haplogroup 2 (Spring 2 to Spring 6), demographic analyses were unable to detect a demographic event in this group. The analysis showed negative and statistically significant values of Fu’s FS neutrality test at the global level (FS = −9.706, *P* = 0.028) and for all populations grouped by haplogroups (Table 3), suggesting a demographic range expansion process. In contrast, the Tajima’s *D*-test did not reveal a significant signal of demographic expansion at the global scale (*D* =  − 0.3599, *P* = 0.423). Signals of population expansion were detected in two populations grouped by haplogroups (Haplogroup 1 and 2; Table 3) using Tajima’s *D*-test. The discrepancy between the *D* and *FS* tests is likely to arise from the decreased statistical power of *D* in detecting significant changes in population sizes (Ramos-Onsins & Rozas, 2002). The mismatch distribution of Haplogroups 1 and Haplogroups 2 suggests a recent history of population expansion, a scenario depicted by a typical unimodal and smooth distribution (Fig. 4). The analysis revealed a non-significant SSD value (SSD = 0.753, *P* = 0.123) that did not refute the demographic model of spatial expansion for the species (Table 3). Likewise, the Harpending’s raggedness index was not significant (rH = 0.045, *P* = 0.354; Table 3). A pattern of demographic population expansion for Haplogroup 1 and Haplogroup 3 was further supported by low and statistically non-significant Harpending’s raggedness index and SSD values (Table 3). The demographic scenario for *H. ascotanensis* obtained through the BSPs suggested a slight to moderate demographic expansion that occurred in the recent past in Haplogroup 1 and Haplogroup 3 (but at different times), and probably due to extremely low genetic diversity in Haplogroup 2, none demographic event was detected. The time of expansion for the Haplogroup 3 (Spring 11) was estimated to have started in the Late Pleistocene (*c.* 30 kya) through the Last Glacial Maximum (LGM, *c.* 20 kya) until the Early Holocene (*c.* 10 kya), after this population expansion the effective population size (Ne) remained constant (Fig. 4). The BSP analysis for Haplogroup 1 showed a constant Ne since the Late Pleistocene and a sudden population expansion was estimated to have started in the Holocene (*c.* 10 kya) (Fig. 4).

**Table 3:** Demographic parameters. Populations expansion and mismatch distribution parameters for *H. ascotanensis*.

Groups	N	Haplotypes	Tajima’s D		Fu’s Fs		Mismatch		
			D	*P* (Ds < Dobs)	F	*P* (Fs < Fobs)	rH	SSD	*τ*
Haplogroup 1	225	19	−1.6347	0.026	−11.7923	0.001	0.1347 n.s.	0.01480 n.s.	1.0175
Haplogroup 2	63	4	−1.4814	0.025	−3.4827	0.001	0.5150	0.00052 n.s.	3.0000
Haplogroup 3	34	13	−1.1822	0.133	−7.5634	0.001	0.0898 n.s.	0.01699 n.s.	1.8125
Total	322	36	−0.3599	0.423	−9.7060	0.028	0.0450 n.s.	0.75329 n.s.	1.9433

**Notes.**

rH Harpending’s raggedness index SSD sum squared deviations *τ* demographic mutation time parameter

The Mantel test revealed a significant positive correlation between genetic distances and linear geographical distances among populations (*r* = 0.6233, *P* <  0.005). Estimations of past migration rates (*M*, mutation-scaled effective immigration rate) indicated that Haplogroup 2 showed higher immigration rates, followed by Haplogroup 3, whereas Haplogroup 1 showed lower rates (Table 4). Immigration rates from Haplogroup 1 and Haplogroup 3 to Haplogroup 2 were 294.6 and 235.0, respectively. When grouping populations using genetic clusters defined by GENELAND analysis, reciprocal migrations rates among clusters I and II (northern populations) were higher than rates with Cluster III (Table 4). In both analysis, closer populations showed higher migrations rates than distant populations.

**Table 4:** Historic gene flow (MIGRATE-n). Results of the migration rate analysis: pairwise population comparisons of population sizes (Theta) and mutation-scaled effective immigration rate (M) under two grouping schemes (haplogroups and genetic clusters).

Group level	Population	Theta [Ne μ]	*M* (m/μ) [+ = receiving population]		
			1, +	2, +	3, +
Haplogroups	Haplogroup 1	0.006604	–	53.2	63.1
	Haplogroup 2	0.001082	294.6	–	235.0
	Haplogroup 3	0.009516	93.0	102.4	–
Genetic clusters	Cluster I	0.005144	–	408.6	71.9
	Cluster II	0.003550	533.6	–	101.6
	Cluster III	0.008096	86.6	80.1	–

### Morphometric analysis

The first PC (78.64%) was mainly related with shell width while PC2 (5.01%) corresponded to changes in the whorls suture depth (Fig. S1). Only in the first and third datasets (samples pooled by springs and genetics clusters, respectively) results of PERMANOVA tests using the first seven PCs revealed significant differences in the Fourier coefficients between groups analysed (Table 5), although principal components analysis of the shell morphology showed that units analysed clearly overlap in the morphospace (Fig. S2). We tested for multivariate normality and no significant deviation was found. Multivariate discriminant analyses based on shell morphologies across every dataset only detected high levels of correct re-assignment in individuals from Spring 11 (Table 5; Table S2). Under the three grouping schemes: samples sorted by springs, haplogroups and genetic clusters, individuals from Spring 11 were identified correctly 64.71%, 70.58% and 67.65% (jackknifed) of the time, respectively.

**Table 5:** Shell outlines differences in individuals from the Ascotán saltpan. Results of a non-parametric multivariate analysis of variance (PERMANOVA) comparing three datasets (samples sorted by springs, haplogroups and genetic clusters). Also, a classification table obtained from LDA for each dataset is presented. Mean percentage of correctly classified individuals and the highest percentage obtained is showed (complete table in Table S2).

Dataset	Groups number	PERMANOVA	Linear discriminant analysis		
			Mean % C.C.	Mean % C.C. (jackknifed)	Highest % C.C.
Springs	12	*F* = 10.09; *p* = 0.0001	37.37	31.84	64.71 (Spring 11)
Haplogroups	3	*F* = 1.253; *p* = 0.2733	55.16	52.81	70.58 (Haplogroup 3)
Genetic Clusters	3	*F* = 2.718; *p* = 0.0497	55.49	53.08	67.65 (Cluster 3)

## Discussion

The results of the current study revealed that *H. ascotanensis* has a high phylogeographic structure, even when its distribution encompass only a few kilometres. Using diverse analysis to examine spatial and temporal patterns of mtDNA sequence variation and morphological differentiation, a complex pattern of historical and demographic events was uncovered in this endemic snail. Three main findings were revealed from the present study: (i) a pronounced mtDNA sequence differentiation at small spatial scale; (ii) complex historical and demographic influences on sequence divergence and diversity within and among springs and; (iii) an incipient morphological differentiation coupled to genetic divergence.

The haplotype network and genetic clusters defined by GENELAND analysis indicate that there are three genetic entities inhabiting the Ascotán saltpan, although the composition of the groups was partially discordant. Both analyses defined specimens from Spring 11 as a different population (Haplogroup 3 and Cluster III), a result congruent with the physiographic arrangement of the saltpan since Spring 11 is the southernmost spring in the system and currently is completely isolated. However, there was an inconsistency in the northern springs’ classification, the haplotype network defined a haplogroup widely distributed in the saltpan (Haplogroup 1), covering all springs except Spring 11. Spatially nested in Haplogroup 1 was defined Haplogroup 2, which mainly grouped samples from Spring 2 to Spring 6. According to the haplotype network, haplogroups 1 and 2 were the most genetically distant groups (at least nine mutational steps). In contrast, GENELAND analysis defined Cluster I as the individuals from Spring 1, Spring 7 to Spring 10, and Spring 12; and Cluster II contained individuals from Spring 2 to Spring 6. The Cluster II probably reflects an influence of geographical proximity among springs in the result, since the analysis classified the most genetic distant individuals into the same population. This was also evidenced by the AMOVAs analyses, which showed that the genetic variance was better explained sorting samples by haplogroups than genetic clusters.

Whilst some springs contained highly similar genetic snails, Spring 2 to Spring 6 were constituted by individuals with very divergent sequences. This pattern suggests that in these springs exist two highly genetically structured groups that probably have different evolutionary histories. We propose two main hypotheses to explain this pattern. The first and most probable hypothesis suggests that occurred an allopatric divergence followed by a range expansion leading to secondary contact among the two genetic groups detected. The second hypothesis proposes a local adaptation accompanied by genetic divergence (i.e., an *in situ* differentiation); but considering that the saltpan was a continuous hydrological system in the past, and that afterwards the passing of humid periods to a dry season the system was divided into isolated or partially isolated springs suggest that this is a less feasible scenario. The range expansion considered in the first hypothesis may be due to dispersal mediated by high rainfalls seasons during SASM (Morales et al., 2012) that could connect springs close enough to allow genetic flow between populations (as proposed by Cruz-Jofré et al., 2016), subterranean hydrological connections undetected or passive dispersal mediated by birds as has been reported in similar freshwater snails from desert hydrological systems in Australia (Van Leeuwen et al., 2012a; Van Leeuwen et al., 2012b; Van Leeuwen et al., 2013). The results suggest that genetic differentiation is not only a response to a physiographic imposition, but also an active reaction to climatic changes, and additionally, modulated by biological properties (such as dispersal capacities) of the organism. This pattern has been described previously in diverse freshwater taxa confined to hydrological systems located in arid regions (Moline et al., 2004; Wilmer et al., 2008; Murphy, Guzik & Wilmer, 2010; Murphy et al., 2015).

Our demographic analyses revealed that *H. ascotanensis* populations present different histories. Population inhabiting the southernmost spring (Spring 11, Haplogroup 3) showed a constant population expansion and growth since *c.* 0.30 kya until *c.* 0.10 kya, whereas northern populations show signals of recent demographic expansion (*c.* 10 kya, Haplogroup 1) and recent demographic stability (Haplogroup 2). Although the exact mechanisms that account for this are unclear, the constant population expansion denoted by BSP analyses in Haplogroup 3 could be related with the last three major humid periods reported in the Altiplano associated with paleolakes Sajsi (*c.* 21–25 kya), Tauca (*c.* 14–18 kya) and Coipasa (*c.* 11–13 kya) (Blard et al., 2009; Blard et al., 2011; Placzek, Quade & Patchett, 2011; Placzek, Quade & Patchett, 2013). Since Spring 11 is located at a greater altitude and distant from other springs, probably it was the first spring to become disconnected in every arid period, therefore its population could possess an independent demographic and evolutionary history, not shared with the other populations of the saltpan. Studies in co-distributed taxa (snails and fishes) suggest that populations from Spring 11 are genetically divergent to the remaining springs (Morales, Vila & Poulin, 2011; Collado & Méndez, 2013; Cruz-Jofré et al., 2016). Under the same argument, haplogroups 1 and 2 probably had a history of their own (this is denoted by the large amount of genetic differences accumulated), however, they are currently geographically nested, probably due to dispersal mediated by flooding occurred in recent climatic events. Even though they share a common habitat, the demographic history of each of these populations is discordant. Haplogroup 2 has a recent demographic stability with extremely low genetic diversity. In contrast, Haplogroup 1 shows signals of a recent population expansion that is not coincident with any major climatic event reported in the Altiplano, so it could be associated with contemporary changes in the rainfall of the region (Morales et al., 2012; Placzek, Quade & Patchett, 2013) or an increase of the genetic diversity as a result of a range expansion that could be associated with the secondary contact proposed above.

Estimates of the population expansion in Haplogroup 3 (Spring 11) corresponded approximately to the transit through three major humid periods (associated with paleolakes Sajsi, Tauca and Coipasa), suggesting that these snails persisted isolated in the saltpan during the LGM, since they have unique haplotypes not present in other springs. The results indicate that the transition from humid to arid periods had apparently little effect on the population size in Haplogroup 3, which is an important finding considering that these strictly aquatic organisms need to cope with an increase in salinity and dehydration, which are *conditio sine qua non* to the saltpan formation. Probably the dynamic change in water availability impose a pressure that promote the development of strategies to respond to a variable environment, and the contrasting demographic histories and genetic diversification suggest that the strategies could be different among the populations of *H. ascotanensis*; similar environmental changes have caused comparable evolutionary responses in analogous hydrological-desert systems (Pfenninger & Posada, 2002; Mossop et al., 2015; Murphy et al., 2012; Murphy et al., 2015).

Morphometrics analyses recovered only one of the three populations defined by the genetic analyses. Samples from Spring 11 were correctly assigned to their corresponding group roughly 70% of the time, a high value considering that these snails present a cryptic morphology (Collado, Valladares & Méndez, 2013; Collado, Valladares & Méndez, 2016). This evidence indicates that snails from Spring 11 have a particular morphology. In other snails, morphological differentiation has been related to multiples causes including ecological and environmental factors (Dillon, 1984; Goodfriend, 1986; Chiu et al., 2002; Guerra-Varela et al., 2007), parasitism (Alda et al., 2010), growth rate (Kemp & Bertness, 1984), predator induced response (Brönmark, Lakowitz & Hollander, 2011) and intraspecific morphological variation associated with geographical isolation (Sánchez et al., 2011; Sepúlveda & Ibáñez, 2012). In *H. ascotanensis*, the morphological differentiation showed by Spring 11 could be induced by genetic differentiation, possibly caused by geographical isolation and adaptive responses to dynamic environmental conditions, as has been reported in the gastropod *Potamopyrgus antipodarum* (Gray, 1843) (Kistner & Dybdahl, 2013). This argument is supported by MIGRATE analyses that indicate that Spring 11 presented low historical genetic flow regarding other springs and the Mantel test suggested an isolation-by-distance pattern.

Finally, our results lend strong support to the hypothesis that species (and populations) respond differently to the same environmental and historical circumstances. Ecological conditions and interactions, and environmental impositions (such as aridity) influence the genetic pool or structure in different taxa, but biological properties (e.g., dispersal capacities, life history traits, inter-specific interactions, etc.) may led to large differences in phylogeographic history (Zickovich & Bohonak, 2007; Murphy, Guzik & Wilmer, 2010; Sproul et al., 2014; Murphy et al., 2015). On the one hand, our study on *H. ascotanensis* compared to other taxa co-distributed in the Ascotán saltpan indicates that species show a deep genetic structure, but the times of divergence and the organization of population is slightly different (Morales, Vila & Poulin, 2011; Collado & Méndez, 2013; Cruz-Jofré et al., 2016). Considering our results and based on previous studies, we can conclude that *Heleobia ascotanensis*, *B. crequii* (gastropod) and *O. ascotanensis* (killifish) present a common pattern in which Spring 11 is genetically divergent from other springs, a result probably related with the geographical isolation of this system. On the other hand, genetic structure in northern springs are idiosyncratic of each taxon, implying that each specie (or population) respond independently to historical changes and that the particular habitat itself is not a sufficient predictor of the genetic differentiation.

## Conclusion

In this study, we provide the first phylogeographical investigation on a species of the genus *Heleobia* from the Southern Altiplano. We present evidence of a high genetic structure in *H. ascotanensis*. Molecular analyses detected three main haplogroups well differentiated and related with the saltpan physiography. Gene flow and demographic analyses showed different connectivity and histories among populations, indicating that each haplogroup responded independently to the fragmentation of the aquatic system occurred during humid-arid transitions that characterized the Plio-Pleistocene period. Morphometric analyses detected a subtle differentiation, and this variation is concomitant with genetic divergence found in Spring 11, the southernmost spring. The genetic structure found in *H. ascotanensis* is similar to the pattern detected in co-distributed taxa; however, temporal and spatial differences among species populations indicate that each taxon (or population) responded differently to the same historic and contemporary climatic changes. Further work in *H. ascotanensis* should focus on increasing analysed loci (besides markers with a uniparental mechanism of inheritance as COI), a strategy that would be helpful for evaluating in detail the mode and tempo of the genetic differentiation detected in this species.

##  Supplemental Information

10.7717/peerj.5802/supp-1 Figure S1 Shell variability of *H. ascotanensis* Shell outlines changes associated with Principal Component 1 (PC1 = 78.64%) and Principal Component 2 (PC2 = 5.01%).

10.7717/peerj.5802/supp-2 Figure S2 Morphometric space of *H. ascotanensis* under three different grouping schemes Principal component (PC) analysis sorting individuals by (A) springs; (B) haplogroups (median-joining network); and (C) genetic clusters (GENELAND analysis). PC1 and PC2 explained 78.64% and 5.04% of the variance, respectively.

10.7717/peerj.5802/supp-3 Table S1 Estimates of pairwise population genetic differentiation Pairwise FST values from mtDNA (COI) analysis are below the diagonal and significance values are above the diagonal.

10.7717/peerj.5802/supp-4 Table S2 Morphometric analysis differentiation and assignment Results of the non-parametric multivariate analysis of variance (PERMANOVA) and linear discriminant analysis (LDA) grouping individuals under three classifications schemes (springs, haplogroups and genetic clusters). For LDA analysis, results are expressed as the percentage of correct classification.

10.7717/peerj.5802/supp-5 Supplemental Information 1 Dataset used for analyses Fasta formatted.

## Additional Information and Declarations

### Field Study Permissions

The following information was supplied relating to field study approvals (i.e., approving body and any reference numbers): Field collections were approved by the Subsecretaría de Pesca y Acuicultura, Ministerio de Economía, Fomento y Turismo, República de Chile (Resolution No. 3285).

### DNA Deposition

The following information was supplied regarding the deposition of DNA sequences: GenBank accession numbers MF447901 to MF448222.

### Data Availability

The following information was supplied regarding data availability: The genetic alignment is available in the Supplemental Files.
