# Supplementary figures and images for: Influenced but not determined by historical events: genetic, demographic and morphological differentiation in Heleobia ascotanensis from the Chilean Altiplano
Source: PeerJ. 2018 Dec 17;6:e5802. doi: 10.7717/peerj.5802 (PMC6301281; doi:10.7717/peerj.5802)

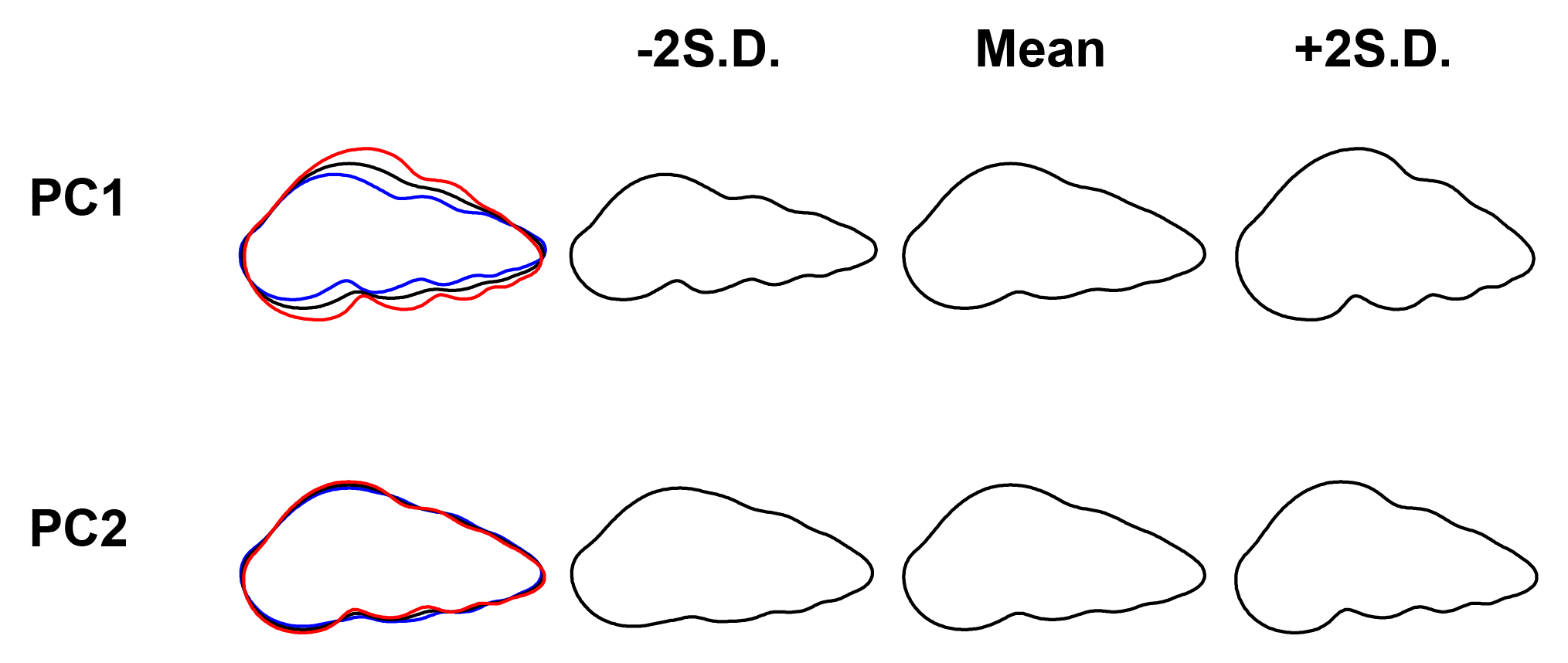

Supplement: Figure S1 — Shell outlines changes associated with Principal Component 1 (PC1 = 78.64%) and Principal Component 2 (PC2 = 5.01%). [file peerj-06-5802-s001.png]

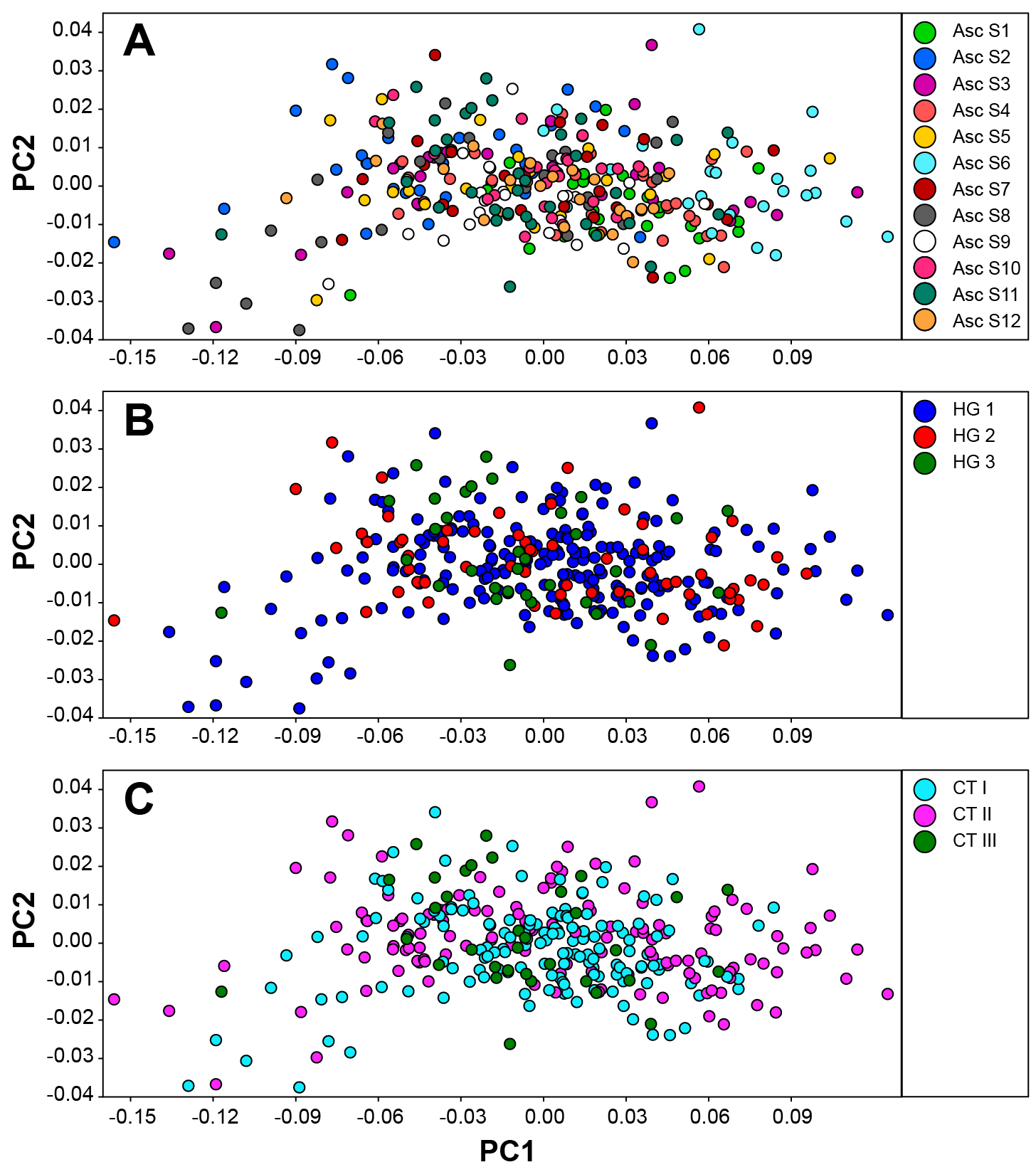

Supplement: Figure S2 — Principal component (PC) analysis sorting individuals by (A) springs; (B) haplogroups (median-joining network); and (C) genetic clusters (GENELAND analysis). PC1 and PC2 explained 78.64% and 5.04% of the variance, respectively. [file peerj-06-5802-s002.png]
