# Supplementary material for: Influenced but not determined by historical events: genetic, demographic and morphological differentiation in Heleobia ascotanensis from the Chilean Altiplano
Source: PeerJ. 2018 Dec 17;6:e5802. doi: 10.7717/peerj.5802 (PMC6301281; doi:10.7717/peerj.5802)
Supplement: Table S1 — Pairwise FST values from mtDNA (COI) analysis are below the diagonal and significance values are above the diagonal. [file peerj-06-5802-s003.docx]

|  | S1 | S2 | S3 | S4 | S5 | S6 | S7 | S8 | S9 | S10 | S11 | S12 |
| --- | --- | --- | --- | --- | --- | --- | --- | --- | --- | --- | --- | --- |
| S1 |  | <0.001 | 0.009 | <0.001 | <0.001 | <0.001 | <0.001 | <0.001 | <0.001 | 0.003 | <0.001 | 0.001 |
| S2 | 0.34571 |  | 0.078 | 0.511 | 0.453 | 0.357 | <0.001 | <0.001 | <0.001 | <0.001 | <0.001 | <0.001 |
| S3 | 0.15064 | 0.06548 |  | 0.037 | 0.124 | 0.224 | <0.001 | <0.001 | <0.001 | <0.001 | <0.001 | <0.001 |
| S4 | 0.39922 | 0.00000 | 0.11316 |  | 0.461 | 0.257 | <0.001 | <0.001 | <0.001 | <0.001 | <0.001 | <0.001 |
| S5 | 0.32320 | 0.00000 | 0.05782 | 0.00000 |  | 0.768 | <0.001 | <0.001 | <0.001 | <0.001 | <0.001 | <0.001 |
| S6 | 0.26148 | 0.00000 | 0.01820 | 0.00611 | 0.00000 |  | <0.001 | <0.001 | <0.001 | <0.001 | <0.001 | <0.001 |
| S7 | 0.15898 | 0.46726 | 0.33092 | 0.52649 | 0.46579 | 0.41128 |  | 0.395 | 0.831 | 0.023 | <0.001 | 0.084 |
| S8 | 0.25651 | 0.48431 | 0.36933 | 0.54052 | 0.48535 | 0.43536 | 0.01122 |  | 0.106 | <0.001 | <0.001 | 0.003 |
| S9 | 0.12010 | 0.44998 | 0.30092 | 0.50965 | 0.44689 | 0.39066 | 0.00000 | 0.04297 |  | 0.067 | <0.001 | 0.149 |
| S10 | 0.06934 | 0.45585 | 0.30110 | 0.51785 | 0.45323 | 0.39496 | 0.08893 | 0.22997 | 0.05124 |  | <0.001 | 0.771 |
| S11 | 0.58474 | 0.40918 | 0.40513 | 0.44649 | 0.44930 | 0.42522 | 0.71932 | 0.73393 | 0.70305 | 0.71535 |  | <0.001 |
| S12 | 0.07680 | 0.46116 | 0.30981 | 0.52482 | 0.46154 | 0.40298 | 0.05950 | 0.20966 | 0.02323 | 0.00000 | 0.72721 |  |
