# Supplementary material for: Influenced but not determined by historical events: genetic, demographic and morphological differentiation in Heleobia ascotanensis from the Chilean Altiplano
Source: PeerJ. 2018 Dec 17;6:e5802. doi: 10.7717/peerj.5802 (PMC6301281; doi:10.7717/peerj.5802)
Supplement: Table S2 — Results of the non-parametric multivariate analysis of variance (PERMANOVA) and linear discriminant analysis (LDA) grouping individuals under three classifications schemes (springs, haplogroups and genetic clusters). For LDA analysis, results are expressed as the percentage of correct classification. [file peerj-06-5802-s004.docx]

| Dataset | Linear Discriminant Analysis | | | |
| --- | --- | --- | --- | --- |
|  | Group | *N* | % C.C. | % C.C. (jackknifed) |
| Springs  PERMANOVA:  F = 10.09; *P* = 0.0001 | Spring 1 | 29 | 27.59 | 20.69 |
|  | Spring 2 | 41 | 45.45 | 36.36 |
|  | Spring 3 | 23 | 4.35 | 0.0 |
|  | Spring 4 | 27 | 48.15 | 40.74 |
|  | Spring 5 | 24 | 0.0 | 0.0 |
|  | Spring 6 | 25 | 64.00 | 52.00 |
|  | Spring 7 | 24 | 45.83 | 33.33 |
|  | Spring 8 | 24 | 29.17 | 29.17 |
|  | Spring 9 | 23 | 34.78 | 21.74 |
|  | Spring 10 | 24 | 45.83 | 45.83 |
|  | Spring 11 | 34 | 67.64 | 64.71 |
|  | Spring 12 | 24 | 41.67 | 37.50 |
|  | Total | 322 | 37.37 | 31.84 |
| Haplogroups  PERMANOVA:  F = 1.253; *P* = 0.2733 | Haplogroup 1 | 225 | 50.69 | 49.77 |
|  | Haplogroup 2 | 63 | 41.27 | 38.09 |
|  | Haplogroup 3 | 34 | 73.53 | 70.58 |
|  | Total | 322 | 55.16 | 52.81 |
| Genetic Clusters  PERMANOVA:  F = 2.718; *P* = 0.0497 | Cluster I | 148 | 52.70 | 50.68 |
|  | Cluster II | 140 | 43.18 | 40.91 |
|  | Cluster III | 34 | 70.58 | 67.65 |
|  | Total | 322 | 55.49 | 53.08 |
